# Metabolic Engineering of *Pseudomonas putida* KT2440 for the Production of *para*-Hydroxy Benzoic Acid

**DOI:** 10.3389/fbioe.2016.00090

**Published:** 2016-11-28

**Authors:** Shiqin Yu, Manuel R. Plan, Gal Winter, Jens O. Krömer

**Affiliations:** ^1^Centre for Microbial Electrochemical Systems (CEMES), The University of Queensland, Brisbane, QLD, Australia; ^2^Advanced Water Management Centre (AWMC), The University of Queensland, Brisbane, QLD, Australia; ^3^Australian Institute for Bioengineering and Nanotechnology (AIBN), The University of Queensland, Brisbane, QLD, Australia; ^4^Metabolomics Australia (Queensland Node), The University of Queensland, Brisbane, QLD, Australia; ^5^School of Science and Technology, The University of New England, Armidale, NSW, Australia

**Keywords:** *Pseudomonas putida* KT2440, *para*-hydroxy benzoic acid, shikimate pathway, metabolic engineering

## Abstract

*para*-Hydroxy benzoic acid (PHBA) is the key component for preparing parabens, a common preservatives in food, drugs, and personal care products, as well as high-performance bioplastics such as liquid crystal polymers. *Pseudomonas putida* KT2440 was engineered to produce PHBA from glucose *via* the shikimate pathway intermediate chorismate. To obtain the PHBA production strain, chorismate lyase UbiC from *Escherichia coli* and a feedback resistant 3-deoxy-d-arabino-heptulosonate-7-phosphate synthase encoded by gene *aroG*^D146N^ were overexpressed individually and simultaneously. In addition, genes related to product degradation (*pobA*) or competing for the precursor chorismate (*pheA* and *trpE*) were deleted from the genome. To further improve PHBA production, the glucose metabolism repressor *hexR* was knocked out in order to increase erythrose 4-phosphate and NADPH supply. The best strain achieved a maximum titer of 1.73 g L^−1^ and a carbon yield of 18.1% (C-mol C-mol^−1^) in a non-optimized fed-batch fermentation. This is to date the highest PHBA concentration produced by *P. putida* using a chorismate lyase.

## Introduction

The aromatic hydroxy acid, *para*-Hydroxy benzoic acid (PHBA) is commonly used in the chemical industry. It is a key component in the manufacturing of liquid crystal polymers (LCP) with high-value applications in the thermoplastics market (Ibeh, 2011). PHBA is also used to prepare paraben preservatives in the cosmetics, food, and pharmaceutical industries. Current production of PHBA is based on petroleum-derived chemicals through the Kolbe–Schmitt reaction. However, the reaction’s requirements for high-temperature/high-pressure conditions, along with the problem of by-product formation (Yoshida and Nagasawa, 2007), make the production of this aromatic compound relatively expensive. The volatility of raw material prices and decreasing availability in the future, together with environmental concerns are driving the development of a renewable and sustainable process for PHBA production (Erickson et al., 2012; Gavrilescu, 2014).

**Figure 1:**
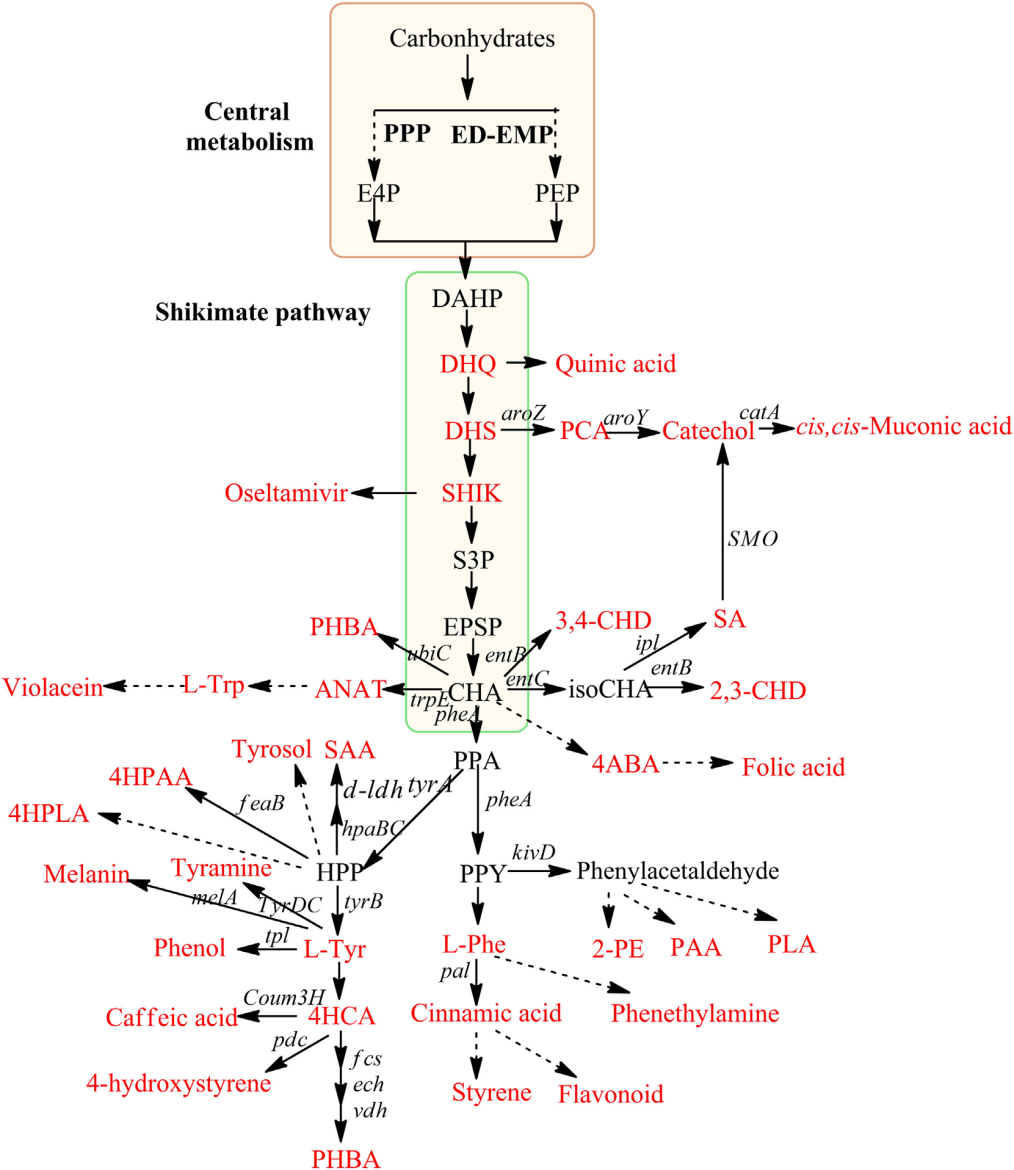
**Production of aromatic compounds via shikimate pathway in microbes**. Red framed box indicates central metabolism, and green framed box indicates shikimate pathway. Compounds have industrial interest are highlighted in red color. Dashed arrows indicate multi-enzymatic synthesis. DHQ, 3-dehydroquinate; DHS, 3-dehydroshikimate; SHIK, shikimate; S3P, shikimate-3-phosphate; EPSP, 5-enolpyruvyl-shikimate 3-phosphate; CHA, chorismate; HPP, 4-hydroxy phenyl pyruvate; ANAT, anthranilate; PPA, prephenate; PPY, phenyl pyruvate; 4ABA, 4-aminobenzoic acid; SAA, salvianic acid A; 4HCA, *p*-hydroxy cinnamic acids; PLA, phenyl lactic acid; 4HPLA, 4-hydroxy phenyl lactic acid; 2-PE, phenyl ethanol; PAA, phenyl acetic acid; 4HPAA, 4-hydroxy phenyl acetic acid; 2,3-CHD, S,S-2,3-dihydroxy-2,3-dihydrobenzoic acid; 3,4-CHD S,S-3,4-dihydroxy-3,4-dihydrobenzoic acid; *aroZ*, 3-DHS dehydratase; *aroY*, PCA decarboxylase; *catA*, catechol 1,2-dioxygenase; *feaB*, phenylacetaldehyde dehydrogenase; *tpl*, Tyr phenol lyase from *Pantoea agglomerans*; tyrDC, tyramine decarboxylase gene from *Lactobacillus brevis*; pdc, 4HCA decarboxylase from *L. plantarum*; *tyrB*, tyrosine aminotransferase; hpaBC, an endogenous hydroxylase from *E. coli*; *ldh*, lactate dehydrogenase from *L. pentosus*; *pal*, Phe-ammonia lyase/Tyr-ammonia lyase from *Rhodosporidium toruloides*; *kivD*, phenylpyruvate decarboxylase tyrA, biofunctional chorismate mutase/prephenate dehydrogenase; entB, isochorismatase; entC, isochorismate synthase; melA, tyrosinase; *fcs, p*-coumaroyl-CoA synthetase; *ech, p*-coumaroyl-CoA hydratase/lyase; *vdh, p*-hydroxybenzaldehyde dehydrogenase; *ipl*, isochorismate pyruvate lyase; SMO, salicylate 1-monoxygenase; *pheA*, bifunctional chorismate mutase/prephenate dehydratase; *tal*, tyrosine ammonia lyase; Coum3H, 4-coumarate hydroxylase; ubiC, chorismate.

Microbial production of chemicals from renewable resources is a sustainable alternative and the bio-production pathways for aromatics and aromatic derivatives have been intensively studied for the past decades (Wierckx et al., 2005; Verhoef et al., 2007; Weber et al., 2012; Krömer et al., 2013; Lin et al., 2014; Averesch et al., 2016). The shikimate pathway is the key pathway for the synthesis of the aromatic amino acids l-tryptophan (Trp), l-phenylalanine (Phe), and l-tyrosine (Tyr), the synthesis of quinones, folates, secondary metabolites, and derived compounds including many commercially valuable compounds (Figure 1) (Gosset, 2009; Karpf and Trussardi, 2009; Koma et al., 2012; Curran et al., 2013; Zhang and Stephanopoulos, 2013). The pathway links carbohydrate metabolism to aromatic compound biosynthesis by converting phosphoenolpyruvate (PEP) and d-erythrose 4-phosphate (E4P) from the central carbon metabolism into 3-deoxy-d-arabino-heptulosonate-7-phosphate (DAHP), after a sequence of seven reactions chorismate, a universal precursor for aromatic amino acids and other aromatic compounds, is formed (Bentley, 1990). Microbial production of PHBA has been studied in engineered strains of *Escherichia coli* (Barker and Frost, 2001), *Klebsiella pneumoniae* (Müller et al., 1995), and in *Saccharomyces cerevisiae* (Krömer et al., 2013; Williams et al., 2015). Barker and colleagues further engineered *E. coli* l-tryptophan-producing strain D2704 by inserting extra copies of genes from shikimate pathway, *aroA* (5-enolpyruvylshikimate 3-phosphate synthase), *aroL* (shikimate kinase), *aroB* (3-dehydroquinate synthase), and *aroF* (feedback-insensitive DAHP synthase) as well as overexpressed *ubiC* (chorismate lyase) and *tktA* (transketolase), achieved a titer of 12 g L^−1^ and a C-mole yield of 13%. Further increase of the yield was unattainable due to the toxic effect this concentration of PHBA has on *E. coli*. Additionally, a significant amount by-product formation was observed in the fermentation and the introduction of many unbalanced genes may lead to metabolic burdens resulting in an undesired fermentation performance.

*Pseudomonas putida* is well known for its versatile metabolism, fast growth rate with simple nutrient requirement, high robustness against harsh environmental conditions, and low cellular maintenance demand. Moreover, *P. putida* has a natural high resistance to PHBA. Few or no by-product formation such as acetate, glycerol, or ethanol which are frequently observed in industrial production host *E. coli, Bacillus subtilis*, and *S. cerevisiae* (Ebert et al., 2011; Poblete-Castro et al., 2012). Furthermore, *P. putida* almost exclusively use Entner–Doudoroff pathway (ED pathway) enabling it generates more NADPH which is important for maximization product titer (Ng et al., 2015) and prevention oxidative stress (Chavarría et al., 2013). The production of shikimate pathway-derived chemicals will benefit from higher NADPH regeneration rate as the conversion of 3-dehydroshikimate into shikimate needs NADPH as the cofactor. All these features not only endow it as a flexible cell factory for bulk chemicals production but also highlight this strain as a promising workhorse for toxic chemicals production. A number of examples demonstrate its great potential in the production of a wide range chemicals, especially toxic aromatics such as phenol (Wierckx et al., 2005), PHBA (Verhoef et al., 2007), 4-hydroxy styrene (Verhoef et al., 2009), 3-nitrocatechol (Prakash et al., 2010), and 4-hydroxy quinaldine (Ütkür et al., 2011). But it is also a suitable host for the production of more traditional bio-products such as poly-hydroxy alkanoates (Le Meur et al., 2012).

Metabolic engineering of *P. putida* for the production of PHBA was first described in strain S12palB1, this strain was able to produce a maximum titer of 12.9 mM PHBA with a C-mole yield of 8.5% on glycerol in a carbon-limited fed-batch fermentation (Verhoef et al., 2007). Yields were further increased by a mixed-substrate feeding strategy (Meijnen et al., 2011). This PHBA production strain was metabolically engineered based on the coumarate pathway (tyrosine degradation, Figure 1), which has a lower theoretical yield than production of PHBA using the chorismate lyase (UbiC) (Krömer et al., 2013). In addition, further improvement of this strain was difficult as the platform strain was based on random nitrosoguanidine (NTG) mutagenesis, and the unknown genetic basis limits strain improvement.

Here, we present the production of PHBA from glucose using the more efficient chorismate lyase route in *P. putida* KT2440 using rational engineering. Increased PHBA production was achieved through genetic deletion of PHBA degrading pathways as well as pathways competing for chorismate. In addition to overexpression of *ubiC*, a feedback-resistant DAHP synthase encoded by *aroG^D146N^* was also overexpressed. Finally, we explored the effect of deletion of the gene encoding for glucose metabolism repressor *hexR* and its effect on PHBA production.

## Materials and Methods

### Bacterial Strains and Plasmids

Bacterial strains and plasmids used in this study are listed in Table 1. *P. putida* KT2440 (DSMZ 6125) was used as production host, and *E. coli* XL10-Gold (Agilent Technologies Inc.) was used to prepare all plasmids except the R6K plasmids which were prepared in *E. coli* DH5α λpir (Martinez-Garcia and de Lorenzo, 2011).

**Table 1:** **The strains and plasmids used in this study**.

	Characteristics	Reference
**Plasmids**		
pSEVA234		Silva-Rocha et al. (2013)
pSEVA234-ubiC	km^R^, expression vector containing the gene of *ubiC*	This study
pSEVA234-UA	km^R^, expression vector containing the gene of *ubiC* and the gene a*roG^D146N^* mutant DAHP synthase	This study
pEMG		Martinez-Garcia and de Lorenzo (2011)
pEMG-*pobA*TS1TS2	km^R^, using for *pobA* gene deletion from genome	This study
pEMG-*pheA*TS1TS2	km^R^, using for *pheA* gene deletion from genome	This study
pEMG-*trpE*TS1TS2	km^R^, using for *trpE* gene deletion from genome	This study
pEMG-*hexR*TS1TS2	km^R^, using for *hexR* gene deletion from genome	This study
**Stains’ name**		
*E. coli* XL10-Gold	*endA1 glnV44 recA1 thi-1 gyrA96 relA1 lac Hte Δ(mcrA)183 Δ(mcrCB-hsdSMR-mrr)173 tet^R^ F΄[proAB lacI^q^ZΔM15 Tn10(Tet^R^ Amy Cm^R^)]*	Stratagene/agilent technologies
*E. coli* DH5α λpir	*endA1 hsdR17 glnV44 (=supE44) thi-1 recA1 gyrA96 relA1 φ80dlacΔ(lacZ)M15 Δ(lacZYA-argF)U169 zdg-232::Tn10 uidA::pir*+	Martinez-Garcia and de Lorenzo (2011)
S0	*Pseudomonas putida* KT2440 DSM 6125, wild type	DSMZ
S1	The stain S0 knockout the gene *pobA* and overexpress gene *ubiC*	This study
S2	S2 derived from strain S1 by the further deletion of *pheA*, overexpress gene *ubiC*	This study
S3	S3 derived from strain S2 by the further deletion of *trpE*, overexpress gene *ubiC*	This study
S4	S4 derived from strain S3 by the further deletion of *hexR*, overexpress gene *ubiC*	This study
S5	S5 derived from strain S3 by the overexpression of *ubiC* and *aroG^D146N^*	This study
S6	S6 derived from strain S4 by the overexpression of *ubiC* and *aroG^D146N^*	This study

*Km^R^, kanamycin resistance*.

### Chemicals and Reagents

All chemicals are of analytical purity, purchased from Sigma-Aldrich (Australia) or Chem-Supply (Australia). Yeast extract and tryptone were purchased from Merck and Oxoid (Australia). Restriction enzymes, T4 ligase, and DNA polymerase were purchased from New England Biolabs (Australia) and were used according to the supplier’s recommendation. Plasmid isolations or DNA fragment purifications were done using GeneJET kits for Plasmid Miniprep, Gel Extraction, or the PCR Purification (Thermo Fisher Scientific Australia Pty. Ltd.).

### Media

Lysogeny broth (LB) consisted of 10 g L^−1^ tryptone, 5 g L^−1^ yeast extract, and 5 g L^−1^ sodium chloride (Lennox, 1955) and was used to culture *E. coli* and *P. putida* strains. Antibiotics of the final concentration of 50 μg mL^−1^ kanamycin or 50 μg mL^−1^ gentamycin were added into medium when it was needed. To make solid plates, 1.5–1.7% (w/v) agar was added into the medium.

Fully chemically defined media were used in shake flask and bioreactor experiments with the following composition: chemically defined medium (CDM) for flask cultivation contained per liter: 6 g Na_2_HPO_4_, 3 g KH_2_PO_4_, 2 g NH_4_Cl, and 0.2 g MgSO_4_⋅7H_2_O and 15 mg CaCl_2_⋅2H_2_O, and 1 mL trace element solution (1.5 g L^−1^ FeCl_3_⋅6H_2_O, 0.15 g L^−1^ H_3_BO_3_, 0.03 g L^−1^ CuSO_4_⋅5H_2_O, 0.18 g L^−1^ KI, 0.12 g L^−1^ MnCl_2_⋅4H_2_O, 0.06 g L^−1^ Na_2_MoO_4_⋅2H_2_O, 0.023g L^−1^ NiCl_2_⋅6H_2_O, 0.12 g L^−1^ ZnSO_4_⋅7H_2_O, 0.15 g L^−1^ CoCl_2_⋅6H_2_O, and 10 g L^−1^ EDTA). This medium was supplemented with 5 g L^−1^ glucose as sole carbon source, 50 μg mL^−1^ kanamycin (where appropriate), 0.5 mmol L^−1^ Trp, and 0.5 mmol L^−1^ phenyl pyruvate (PPY).

Based on Sun’s protocol two different media were used for the seed culture and the fermentation in bioreactor experiments (Sun et al., 2006). Inoculum was prepared in seed medium containing the following components per liter: 4.7 g (NH_4_)_2_SO_4_, 0.24 g MgSO_4_⋅7H_2_O, 6.35 g Na_2_HPO_4_, 2.7 g KH_2_PO_4_, 9.0 g glucose, 15 mg CaCl_2_⋅2H_2_O, 0.75 g tryptone, 0.3 g NaCl and 0.15 g yeast extract, 1 mL trace metal solution, 0.5 mmol Trp and 1 mmol PPY, and 50 mg^−1^ kanamycin. CDM was modified for bioreactor initial fermentation containing per liter: 4.7 g (NH_4_)_2_SO_4_, 0.24 g MgSO_4_⋅7H_2_O, 9.53 g Na_2_HPO_4_, 4.05 g KH_2_PO_4_, 1 g glucose, 15 mg CaCl_2_⋅2H_2_O, 1 mL trace metal solution, 0.5 mmol Trp, and 1 mmol PPY. Feed solution contained per liter: 600 g glucose, 6 g MgSO_4_⋅7H_2_O, 150 mg CaCl_2_⋅2H_2_O, 10 mmol Trp, 100 mg kanamycin, and 10 mL trace metal solution. Phenylpyruvate was fed by syringe based on 0.307 mmol PPY g_CDW_^−1^. The PPY demand was calculated based on the abundance of Phe and Tyr in the *E. coli* biomass composition (Pramanik and Keasling, 1997), since both bacteria belongs to Gammaproteobacteria.

### Cultivation Conditions

All shake flask incubation was carried out in baffled Erlenmeyer flasks in an incubator (2.5 cm orbit, Multitron, Infors, Bottmingen, Switzerland) at 200 rpm. Incubation temperature for flasks and plates was 37°C for *E. coli* and 30°C for *P. putida*.

To select best performing strain, the PHBA production strains were first cultivated for 8 h in 5 mL LB. Fifty microliters of this culture was inoculated into a fresh 25-mL CDM medium overnight, and the next day was inoculated to 35-mL CDM medium in a 250-mL baffled flask to the initial OD_600_ of 0.2. The inducer isopropyl β-d-1-thiogalactopyranoside (IPTG) was added to a final concentration of 1 mmol L^−1^ when the OD_600_ reached 0.3–0.4. Two additional feeds to the final concentration of 0.5 mM PPY and 5 g L^−1^ glucose were added at 6 and 20 h, respectively. Samples were centrifuged (16,000 *g*, 10 min, room temperature) and supernatants were stored in the freezer (−20°C) for further analysis.

Fed-batch fermentations with *P. putida* were carried out in two 1.0 L working volume Biostat B+ bioreactors (Sartorius, Germany), with initial volumes of 0.4 L. Temperature was set at 30 ± 0.5°C and pH was automatically controlled at 7.0 ± 0.1 using 14–15% (w/v) NH_4_OH, which also served as additional nitrogen source for cell growth. Dissolved oxygen (DO) was maintained at a minimum of 40% through automatic control of the agitation between 500 and 2000 rpm followed by variation of air flow from 0.15 to 1.5 L min^−1^. Seed cultures were grown in seed medium at 30°C, 200 rpm flasks overnight (around 13–16 h), collected in mid-exponential phase and then inoculated into the fermenter with initial OD_600_ of 1.0 ± 0.1. The inducer IPTG was added in the early exponential phase (3–4 h), with an initial concentration of 2.5 mM. The feed phase was started half an hour after inoculation (Figure S3 in Supplementary Material). The antifoam polypropylene glycol P2000 (Sigma-Aldrich, Australia) was added when onset of foaming was observed.

### DNA Manipulation

#### Gene Deletions (*ΔpobA, ΔpheA, ΔtrpE*, and *ΔhexR*)

Gene deletions were performed in *P. putida* following a multiple, seamless, and marker-less genomic editing method from Martinez-Garcia and de Lorenzo (2011). The resulting strains are listed in Table 1, and gene knockout process or overexpression is shown in Figure S1 in Supplementary Material. Primers used in this study are listed in Table S2 in Supplementary Material. The pEMG-based knockout plasmids were constructed using a standard restriction and ligation approach. To ease the constructions, the nested fusion PCR approach was applied to increase PCR amplification specificity (Figure S1A in Supplementary Material). Briefly, the fusion gene fragments and pEMG vector were digested with *Bam*HI/*Eco*RI, ligated with a T4 DNA ligase, and transformed into chemical competent cells (*E. coli* DH5α λpir). The positive transformants were obtained from blue–white screening, and further verified with colony PCR, restriction digestion, and sequencing (performed by Australian Genome Research Facility Ltd.), resulting in the four knockout plasmids pEMG-*ΔpobA*, pEMG-*ΔpheA*, pEMG-*ΔtrpE*, and pEMG-*ΔhexR*. The plasmids were electro-transformed into *P. putida*-competent cells according to Choi’s protocol (Choi and Schweizer, 2006). The resulting strains were transformed with the plasmid expressing ISce-I endonuclease (pSW-2) for cutting the pEMG vector backbone (Figure S1B in Supplementary Material). Then, the strains were induced with 15 mM 3-methylbenzoate for 6–8 h, and spread on LB plates containing 50 μg mL^−1^ gentamycin. Finally, selected mutant strains were confirmed using colony PCR with primers of P2/P5. The desired mutant should have the expected size of around 1000 bp (Table S2 in Supplementary Material). Auxotrophic mutants were also verified on solid CDM medium plate, 5 g L^−1^ glucose with and without 0.5 mM Trp, and/or 0.5 mM Phe and 0.5 mM Tyr supplementation (the deletion of gene *trpE* generated the tryptophan auxotroph, and the deletion of gene *pheA* yielded tyrosine and phenylalanine double auxotrophic mutants).

### Expression Plasmid Construction

The genes *ubiC* encoding chorismate lyase from *E. coli* K-12 substr. W3110 (CAA40681.1) and *aroG^D146N^* encoding feedback insensitive DAHP synthase (Kikuchi et al., 1997) were codon optimized and synthesized by Integrated DNA Technologies, Inc. These two genes were digested with *Eco*RI, *Sal*I, and *Hin*dIII, respectively, then inserted into the vector pSEVA234 (Silva-Rocha et al., 2013), resulting in the plasmids pSEVA234-*ubiC* and pSEVA234-UA (Figure S2C in Supplementary Material). The sequencing-verified plasmids were electro-transformed into each of the knockout mutant *P. putida* strains.

### Sodium Dodecyl Sulfate Polyacrylamide Gel Electrophoresis

Culture samples for sodium dodecyl sulfate polyacrylamide gel electrophoresis (SDS-PAGE) were collected by centrifugation (16,000 *g*, 5 min, and room temperature). Pellets were washed with buffer once and resuspended with 50 mM Tris–HCl buffer (pH 7.5). Cells were then disrupted using sonication for 5–10 cycles of 10 s pulse with 10 s interval on ice to prepare protein extracts. For preparation of whole-cell protein samples, the loading buffer was added directly to the crude lysate. For the analysis of soluble protein expression, the cell lysate was first centrifuged at 16,000 *g* for 20 min at 4°C to remove cell debris and insoluble particles. Then, the loading buffer containing β-mercaptoethanol and SDS was added, and heated (5 min, 95–99°C) to denature the protein. SDS-PAGE was performed using 12% polyacrylamide gel according to Laemmli’s protocol (Laemmli, 1970) in a vertical slab gel apparatus from Bio-Rad Laboratories. Coomassie brilliant blue G-250 was used for gel staining.

### Analytical Methods

*Pseudomonas putida* cell densities were monitored by a spectrophotometry (GENESYS 10S UV–Vis, USA) at the wavelength of 600 nm (OD_600_) against water. The correlation between OD and cell dry weight (CDW) was empirically determined to be CDW (g L^−1^) = 0.486 OD_600_. Sugars, organic acids (Lai et al., 2016a), and aromatic compounds (Lai et al., 2016b) were measured by high-performance liquid chromatography (HPLC). Carbon dioxide was monitored by gas analyzer (Balzers Thermostar GSD 300 T3, Liechtenstein). Carbon balance calculation was following the equation of F(C, in) = F(C, out) ≈ F(C, sugars) + F(C, organic acids) + F(C, aromatics) + F(C, CO_2_) + F (C, biomass). Specifically, carbon composition in *P. putida* KT2440 biomass is 48.8% (W W^−1^) (van Duuren et al., 2013).

## Results and Discussion

### Metabolic Engineering of PHBA Production Strains

**Figure 2:**
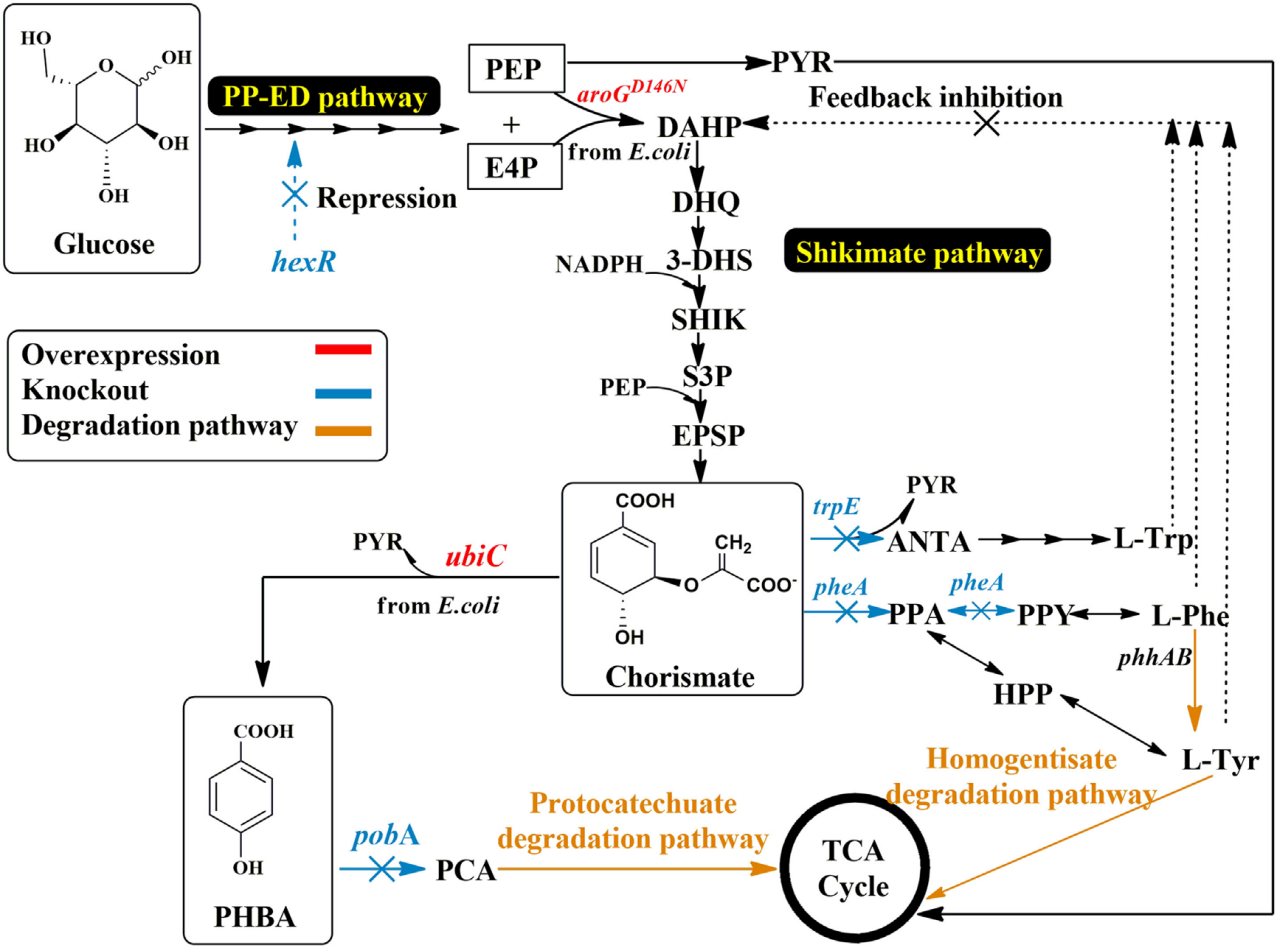
**Schematic view of metabolic engineering in *P. putida* for PHBA production**. AroG^D146N^, feedback resistant DAHP synthase; E4P, erythrose 4-phosphate; HPP, 4-hydroxyphenyl pyruvate; PEP, phosphoenolpyruvate; ANAT, anthranilate; PheA, chorismate mutase/prephenate dehydratase; PhhAB, phenylalanine hydroxylase; PPA, prephenate; PPY, phenylpyruvate; PobA, *p*-hydroxy benzoate hydroxylase; TrpE, anthranilate synthase component I; UbiC, chorismate lyase; HexR, repressor for glucose metabolism.

There is a solid body of research for the metabolic engineering of shikimate pathway-derived aromatics, mainly using *E. coli* and *Corynebacterium glutamicum*. *P. putida* strain development for the production of PHBA can be built on this knowledge and apply successful genetic engineering strategies. Knockout targets were chosen based on their ability to degrade PHBA or to compete with the enzyme responsible for PHBA production (UbiC) for precursor availability. Compared to *E. coli* and *C. glutamicum*, more unique to *P. putida* strains is the ability for aromatics degradation and the multitude of degradation pathways for different aromatics (Jiménez et al., 2002). To increase PHBA concentration, the genes *pobA, pheA, trpE*, and *hexR* were deleted from the genome of *P. putida* KT2440 using the pEMG/pSW-2 system (Martinez-Garcia and de Lorenzo, 2011). The *pobA* gene encodes *p*-hydroxy benzoate hydroxylase and causes product degradation. The gene *pheA* encodes the bifunctional enzyme of prephenate dehydratase and chorismate mutase, this will direct carbon flux into the tyrosine and phenylalanine synthesis, and is a major competitor for the precursor chorismate. The disruption of *trpE*, which encodes anthranilate synthase component I, is likely to reduce the drain of chorismate into the tryptophan biosynthesis. As a consequence, the deletion of *pheA* and *trpE* was carried out to increase precursor supply to PHBA production by blocking the branching of the flux toward the synthesis of the aromatic amino acids (Figure 2). Both deletions resulted in aromatic amino acids auxotrophic mutants, but when depleted would cause the natural feedback inhibition of the shikimate pathway to be alleviated. It is also important to note that *Pseudomonas* species can degrade Phe and Tyr through the homogentisate pathway, which can cause a difficulty to screen Δ*phe* mutant and the inconvenience in fed-batch fermentation. Phe is first converted into Tyr by the action of the gene products of *phhAB* phenylalanine hydroxylase, which are regulated by the PhhR regulator protein in the presence of the two amino acids (Herrera and Ramos, 2007). They are then further degraded *via* the homogentisate degradation pathway, and metabolites are channeled to the tricarboxylic acid (TCA) cycle (Arias-Barrau et al., 2004). Glucose metabolism repressor *hexR* controls glucokinase (*glu*), glyceraldehyde-3-phosphate (*gap-1*), and several Entner–Doudoroff pathway enzymes (*zwf, pgl, edd*, and *eda*), the deletion of this repressor may increase the flux toward pentose phosphate pathway (PPP) and NADPH pool. The deletion of hexR was expected to increase availability of shikimate pathway precursors E4P and the necessary reducing equivalents NADPH (Figure 2). Successful deletants were chosen based on LB screening with the appropriate antibiotic selection as described in Section “Materials and Methods.”

Chorismate lyase encoded by *ubiC* is a key enzyme in PHBA synthesis; it specifically hydrolyzes chorismate into the products PHBA and pyruvate. The *ubiC* gene from *E. coli* K-12 strain was cloned into vector pSEVA234 regulated with Ptrc/LacI^q^. *E. coli* wild-type UbiC enzyme was shown to be insoluble when overexpressed in *E. coli*, protein manipulations made to increase solubility resulted in some loss of enzyme activity (Stover et al., 2000; Holden et al., 2002). To optimize expression of soluble UbiC, different culture temperature (25 and 30°C) and induction time (6 and 8 h) were performed. However, unlike previous work in *E. coli*, we did not observe protein precipitation in *P. putida* in any of the tested conditions. Instead, over 90% of *E. coli* wild type UbiC was present in soluble form in *P. putida* KT2440 using culture condition as described above (Figure S2 and Table S1 in Supplementary Material).

Finally, to further improve the production of PHBA in *P. putida*, the gene encoding the feedback insensitive 3-deoxy-d-arabino-heptulosonate 7-phosphate (DAHP) synthase isoenzyme mutant AroG^D146N^ (Kikuchi et al., 1997) was constructed in the same operon with UbiC, regulated by the Ptrc/LacI^q^ system (Figure S1C in Supplementary Material). This caused an unregulated influx of carbon into the shikimate pathway.

On a side note, it was earlier postulated by Kuepper et al. (2015) after screening more than a thousand colonies for *pheA* knockout mutants on complex medium that the presence of the homogentisate degradation pathway might hamper the positive identification of positive colonies and the addition of precursor PPY in minimal medium was helpful to screen for this mutant (Kuepper et al., 2015). We successfully identified *pheA* mutants on rich medium when looking for tiny colonies that could be easily overlooked amongst the big colonies that did not contain the knockout.

### Evaluation of the PHBA Production Strains in Shake Flasks

**Figure 3:**
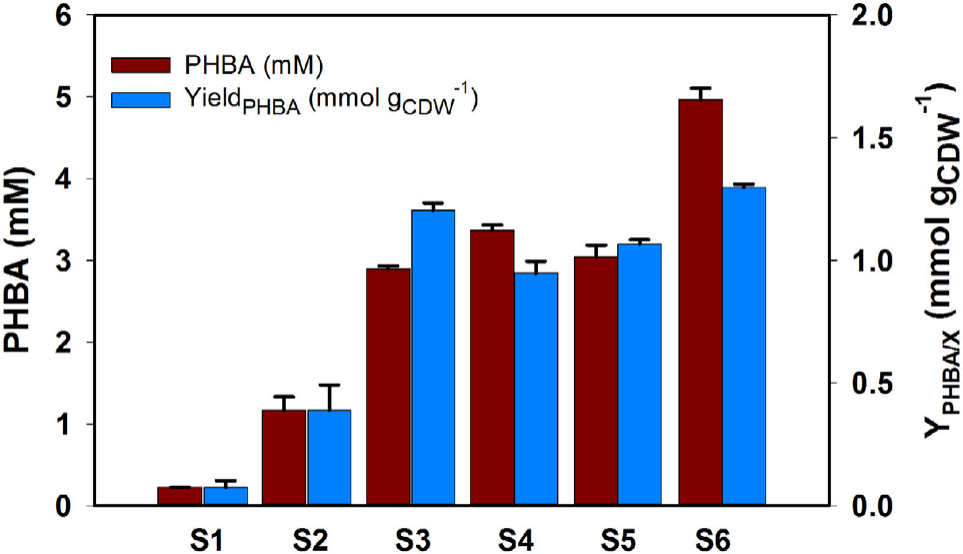
**Various *P. putida* KT2440 mutant strains for PHBA production in shake flask**. All mutants were grown in CDM medium supplemented with 5 g L^−1^ glucose and phenyl pyruvate and tryptophan as described above. Two additional feeds of 5 g L^−1^ glucose and 0.5 mM PPY were added after 6 and 20 h, respectively. All data were from triplicate biological repeats at the late stationary time point of 44 h. Genotypes for mutants tested in this study. S1, *KTΔpobA/234-ubiC*; S2, *KTΔpobApheA/234-ubiC*; S3, *KTΔpobApheAΔtrpE/234-ubiC*; S4, *KTΔpobApheAΔtrpEΔhexR/234-ubiC*; S5, *KTΔpobApheAΔtrpE/234-ubiC-aroG^D146N^*; and S6, *KTΔpobApheAΔtrpEΔhexR/234-ubiC-aroG^D146N^*.

The performance of the genetically engineered strains described above (S1–S6, Table 1) was evaluated for PHBA production in shake flasks, showing a maximum yield of 1.30 ± 0.01 mmol_PHBA_ g_CDW_^−1^, a titer of 4.96 ± 0.146 mM, and 7% (C-mol C-mol^−1^) (Figure 3). The single knockout strain containing the over expression plasmid of *ubiC* (strain S1) produced 0.23 mM PHBA with a yield of 0.075 mmol_PHBA_ g_CDW_^−1^. An increase of around fivefold in titer and yield was achieved with the deletion of chorismate precursor competing genes *pheA* (strain S2). Compared to S1, this was further increased to 12- and 16-fold increase in titer and yield, respectively, by the deletion of the second precursor competing gene *trpE* (strain S3). Overall, indicating a positive effect when deleting precursor competing genes. The knockout of *hexR* in the S3 background did not lead to a very large increase in yield and titer (strain S4) nor did the overexpression of the feedback resistant DAHP synthase (strain S5). However, when combining the knockout of *hexR* with overexpression of DAHP synthase (strain S6) a remarkable increase of 22- and 17-fold increase in titer and yield was achieved relative to the base strain. Compared to S3, S4 has no significant increase in yield and titer, and it can be explained by the fact that our limited feeding of phenylpruvate and tryptophan alleviated or diminished feedback inhibition to their native DAHP synthase in *P. putida*. Unexpectedly, compared to S3, the *hexR* knockout has no substantial improvement for PHBA production but the hexR knockout strain combined overexpression of DAHP synthase, has a significant improvement in titer and yield. The possible reason that the native DAHP synthase was not strong enough to drain flux from central metabolism, interestingly, the combination of both genetic manipulations can synergistically drain the flux from central metabolism to shikimate pathway for the PHBA production, leading to a significant improvement either in titer or in yield. The reason for the increase could be that *Pseudomonas*, like many bacteria, features an imbalanced supply of PEP and erythrose 4-phosphate (E4P), and increasing PPP flux was demonstrated to increase aromatic production in several bacteria including *P. putida* (Chassagnole et al., 2002; Fuhrer et al., 2005; Nikel et al., 2015). The general strategies to increase the E4P supply are to express genes from PP pathway such as *tktA* encoding transketolase and/or *tal* encoding transaldolase (Lu and Liao, 1997; Ikeda and Katsumata, 1999). The role of the hexR protein as a transcriptional repressor of *zwf* encoding the committing step into PPP was described in *P. putida* and its deletion led to increased flux of PPP and reduction of the NADP^+^ pool (del Castillo et al., 2008; Meijnen et al., 2012). In our study, titer increased significantly in the S6 strain, likely due to an increased precursor supply for our PHBA production from glucose.

### PHBA Production by Fed-Batch Fermentation

**Figure 4:**
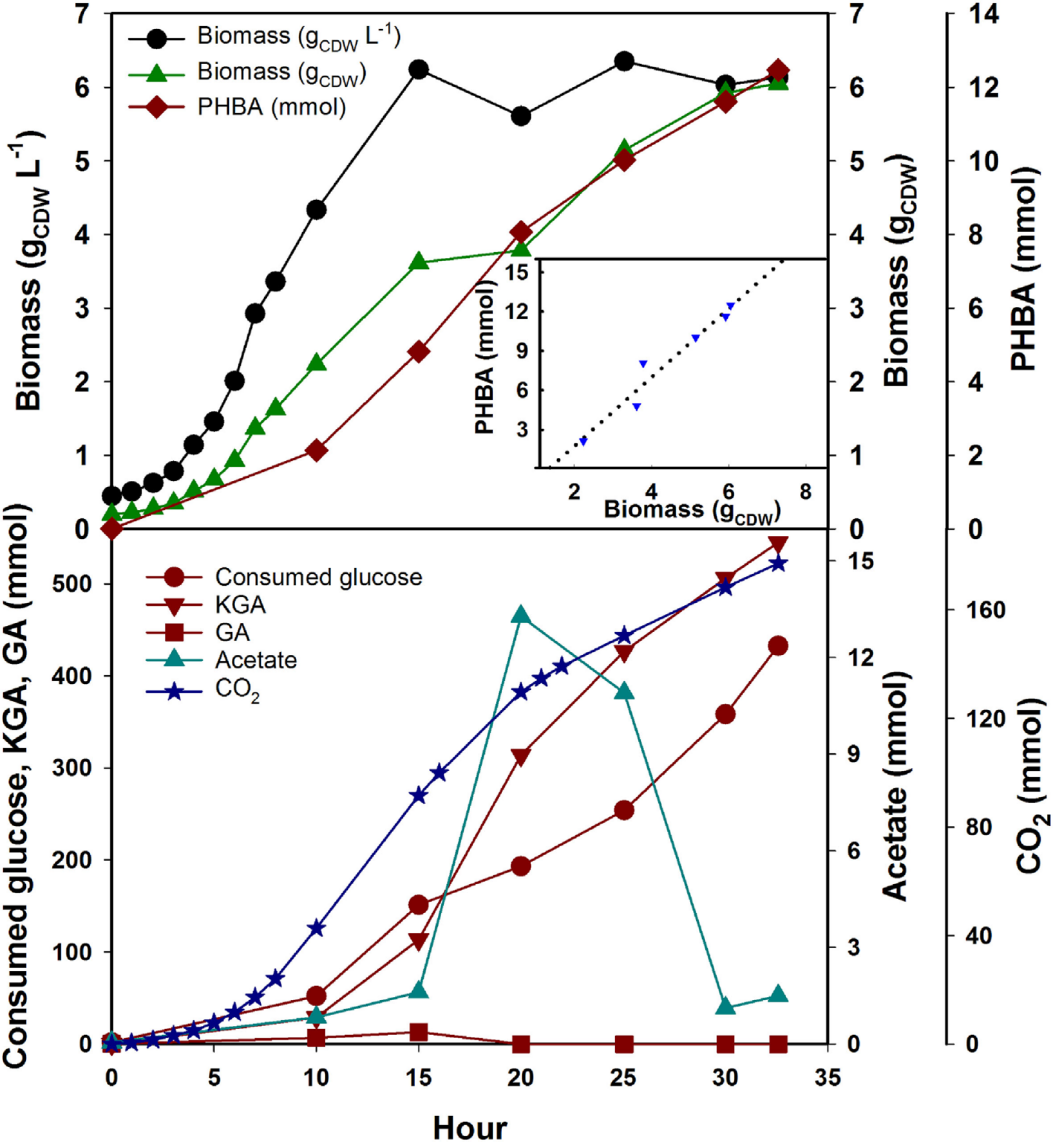
**Profile of mutant strain *KT*Δ*pobA*Δ*pheA*Δ*trpE*Δ*hexR*/234UA in phenyl pyruvate limited fed-batch fermentation in a controlled reactor**. 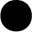, biomass, g_CDW_ L^−1^; 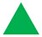, biomass (gCDW); 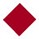, PHBA (mmol); 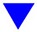, PHBA yield (mmol g_CDW_^−1^); 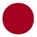, consumed glucose (mmol); 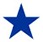, CO_2_ (mmol); 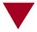, KGA, keto-gluconate (mmol); 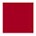, GA, gluconate (mmol); 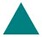, acetate (mmol). Insert is showing the yield coefficient (slope of dashed line = 2.6 mmol_PHBA_ g_CDW_^−1^).

The best PHBA producing strain (S6) was selected from the flask experiment to evaluate its potential in a fed-batch fermentation. *P. putida* will quickly degrade the provided aromatic amino acids Phe and Tyr in fermentation due to the homogentisate degradation pathway. This would lead to growth arrest in our Δ*pheA* strains, or the need for a constant feed of those amino acids. Instead of feeding the two amino acids, we fed the precursor PPY. This feed did not stop degradation through the homogentisate pathway completely but slowed down the process. We assumed *P. putida* KT2440 has a similar biomass composition to *E. coli*, since both species are γ-proteobacteria (Blank et al., 2008) and calculated the necessary PPY feed based on the demand for Tyr and Phe. Glucose concentration was 1 g L^−1^ at the start of the fermentation and was fed as described above after inoculation. PPY solution was fed with 0.307 mmol g_CDW_^−1^ based on the actual cell density at several intervals (5, 8, 10, 15, 20, and 25 h). Biomass kept growing after OD_600_ reached ~12 and 6.05 g CDW were obtained after 32 h. Fermentation was stopped when the working volume of the reactor was reached. Due to the volume change in the fed-batch the following section will discuss absolute amounts. The carbon balance closed at 100.6% meaning that all carbon could be accounted for. As the major by-products, α-keto-gluconate was accumulated (545 mmol) and CO_2_ was released (177 mmol) and about half the fed glucose (~765 mmol) remained unused. This was due to the fact that glucose was not limiting in the fed-batch but PPY instead. *P. putida* will oxidize glucose in periplasm to gluconate and then to α-keto-gluconate. In future experiments, the feed regime should be optimized. Nevertheless, PHBA accumulated over time and reached a maximum titer of 12.46 mmol (12.5 mM at the end of fermentation), and a yield of 2.6 mmol PHBA g_CDW_^−1^ (Figure 4). The product on substrate yield of PHBA based on the consumed C_6_ molecules and PPY (excluding extracellular glucose and α-keto-gluconate, which were not taken up by the cells), reached 18.1% (C-mol C-mol^−1^). Smaller amounts of pyruvate, lactate, and succinate could also be observed to accumulate throughout the fermentation (data not shown). The only other major by-product was acetate at 13 mmol which accumulated in the first 20 h and was consumed thereafter (Figure 4). This might indicate a redirection of intracellular fluxes around the 20-h mark, but this remains to be studied in more detail.

In summary, the fed-batch process was able to achieve higher carbon yields than the shake flask experiments, but the feeding still requires optimization. Compared to published results we achieved a similar titer but a higher yield 18.1% (C-mol C-mol^−1^). *P. putida* S12palB1 (8.5 C-mol C-mol^−1^) was engineered based on the coumarate pathway and had optimized carbon flux toward l-tyrosine (Verhoef et al., 2007), and *P. putida* S12pal_xylB7 (16.3 C-mol C-mol^−1^) used an optimized mixed-substrate feeding strategy and also the coumarate pathway (Meijnen et al., 2011). While the experimentally observed yields are not yet approaching the calculated theoretical yields of the pathways (Krömer et al., 2013), this might still indicate that the UbiC based route has a higher potential for biotechnological production of PHBA.

## Conclusion

*Pseudomonas putida* strains show high potential for industrial aromatic production, mainly due to their high tolerance toward toxic aromatics and the reduced formation of by-products. In this paper, we described *P. putida* production of PHBA from glucose as sole carbon source *via* the chorismate lyase from *E. coli*, which has a higher theoretical yield than the previously described coumarate pathway. The mutant strain *KTΔpobAΔpheAΔtrpEΔhexR/*234UA achieved a maximum titer of 12.5 mM in a PPY limited fed-batch fermentation. This was achieved using a stable, scar-less, and marker-less deletion method, to meet industrial application requirements. This study lays the basis for using *P. putida* as a PHBA production host. Further metabolic engineering strategies can be applied to enhance production, for example, balancing the precursors PEP and E4P or increasing the availability of the reducing equivalent NADPH.

## Author Contributions

SY performed the experiments, designed the study, analyzed data, and drafted the manuscript. MP performed quantitative analytics. GW co-supervised the study and edited the manuscript. JK designed and supervised the study, analyzed data, and edited the manuscript. MP performed HPLC operation, and JO and SY performed carbon balance analysis.

## Conflict of Interest Statement

The authors declare that the research was conducted in the absence of any commercial or financial relationships that could be construed as a potential conflict of interest.
