# Supplementary material for: Metabolic Engineering of Pseudomonas putida KT2440 for the Production of para-Hydroxy Benzoic Acid
Source: Front Bioeng Biotechnol. 2016 Nov 28;4:90. doi: 10.3389/fbioe.2016.00090 (PMC5124731; doi:10.3389/fbioe.2016.00090)
Supplement: Supplementary file 1 [file data_sheet_1.pdf]

Metabolic engineering of *Pseudomonas putida* KT2440 for the production of para-hydroxy benzoic acid

Shiqin Yu[a,b], Manuel Plan[c,d] , Gal Winter[a,b,e] ,Jens O. Krömer[a,b*]

Supplementary material

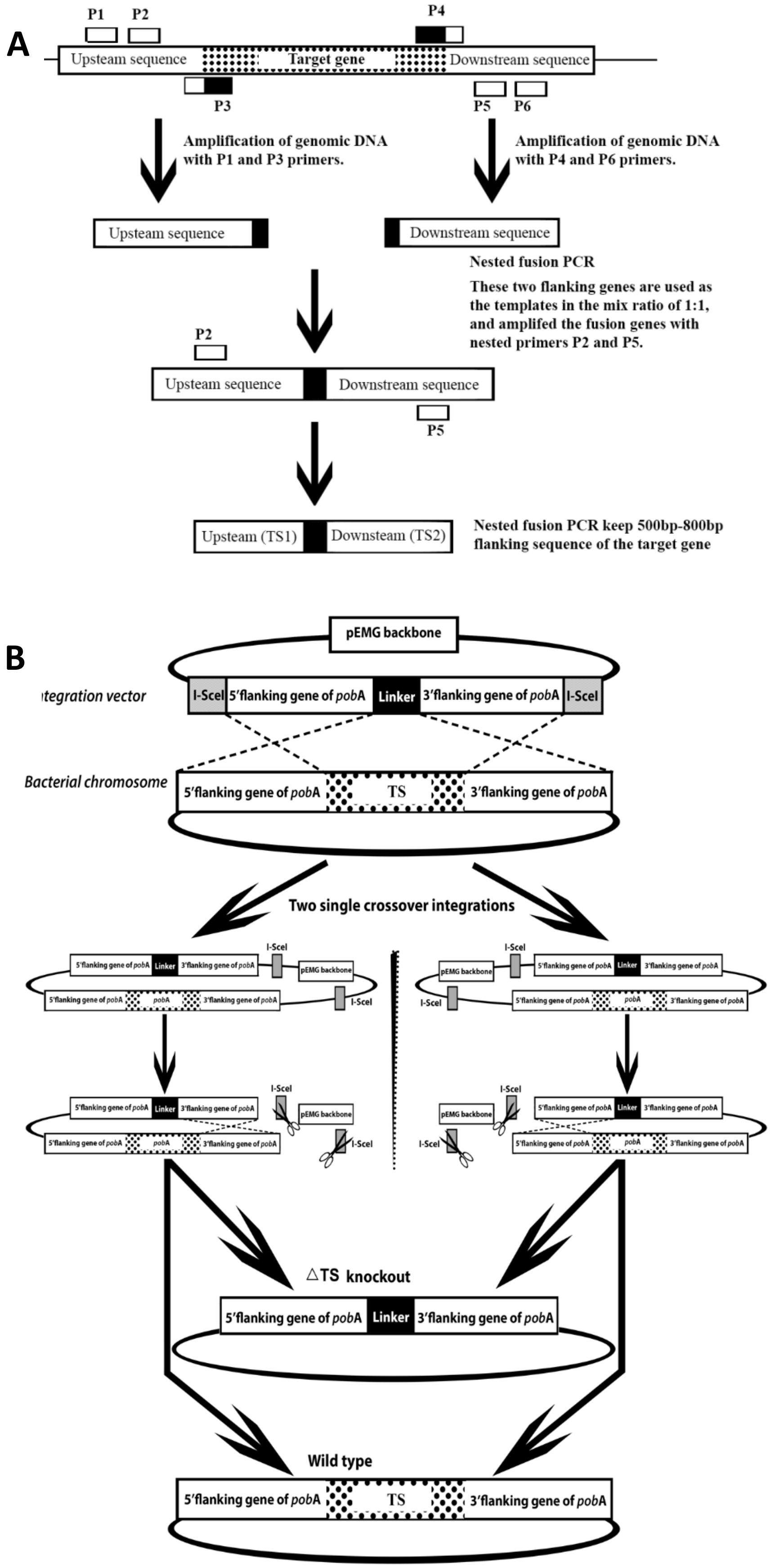
A
P1
P2
P4
Upsteam sequence
Target gene
Downstream sequence
P3
P5
P6
Amplification of genomic DNA with P1 and P3 primers.
Amplification of genomic DNA with P4 and P6 primers.
Upsteam sequence
Downstream sequence
Nested fusion PCR
These two flanking genes are used as the templates in the mix ratio of 1:1, and amplifed the fusion genes with nested primers P2 and P5.
P2
Upsteam sequence
Downstream sequence
P5
Upsteam (TS1)
Downsteam (TS2)
Nested fusion PCR keep 500bp-800bp flanking sequence of the target gene
B
pEMG backbone
ıtegration vector
I-SceI
5′flanking gene of pobA
Linker
3′flanking gene of pobA
I-SceI
Bacterial chromosome
5′flanking gene of pobA
TS
3′flanking gene of pobA
Two single crossover integrations
△TS knockout
5′flanking gene of pobA
Linker
3′flanking gene of pobA
Wild type
5′flanking gene of pobA
TS
3′flanking gene of pobA

Fig.S1 markerless genomic editing tools for *Pseudomonas putida*

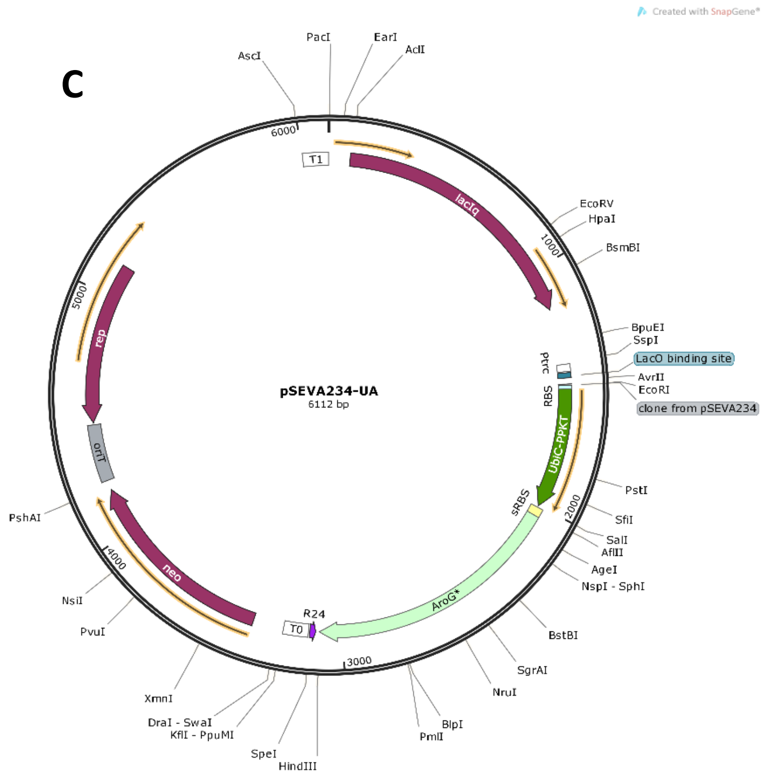

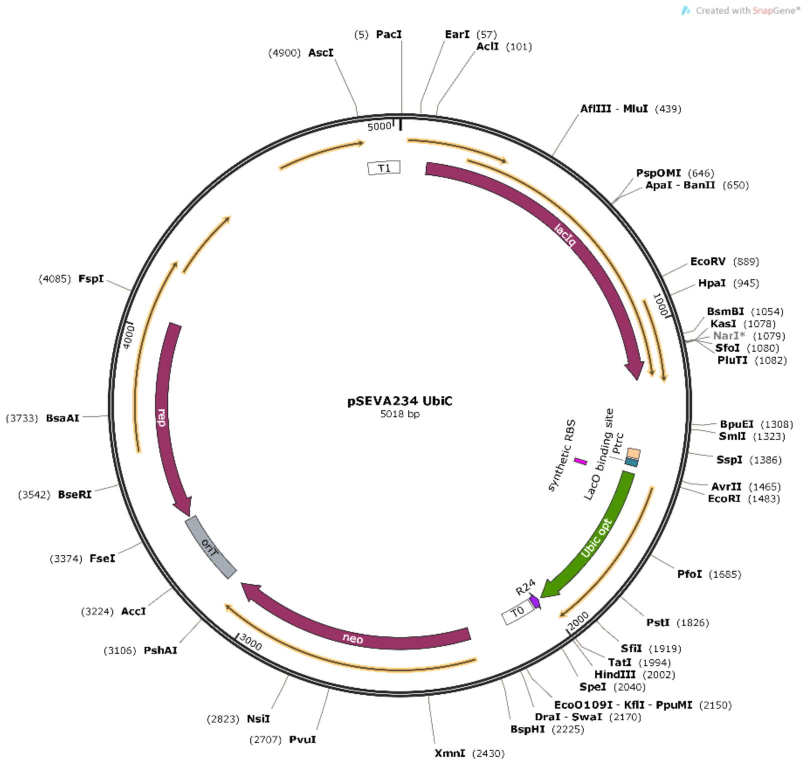

Figure S1. Molecular tools for strain development. (A) The design of nested fusion PCR for the construction of pEMG-TS1TS2. (B) The procedure for gene deletion from genome. (C) Overexpression UibC and / or *aroG*^{D146N} in the pSEVA234 vector using the Ptrc/lacI^{Q} system

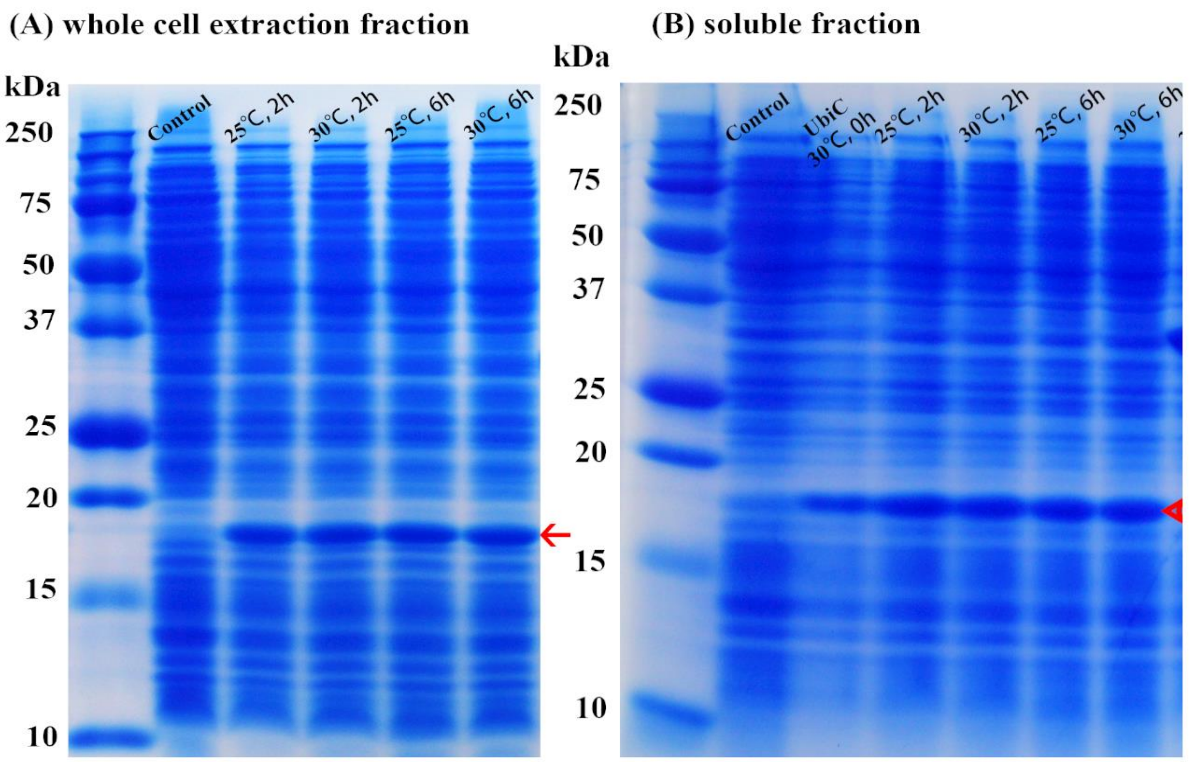

Figure S2. SDS-PAGE analysis of soluble expression of wild type UbiC in *P. putida* KT2440. All cultures were performed in CDMF medium with 5 g L^{-1} glucose as the carbon source, induced with 1mM IPTG when OD600 reach to 0.3-0.4. The control is the strain containing empty vector pSEVA234. Red arrow indicate target protein UbiC. Soluble protein amount was quantified by Pierce BCA Protein Assay Kit, and loading around 40 µg for each sample.

Table S1. Comparison of soluble fraction and whole cell expression of wildtype UbiC

Culture conditions	ratio of soluble fraction to the whole extraction fraction
25℃, 2h	87.9%
30℃, 2h	93.5%
25℃, 6h	91.4%
30℃, 6h	96.3%

Note: The expression level of UbiCwere quantified using the software of Quantity One (Bio-rad).

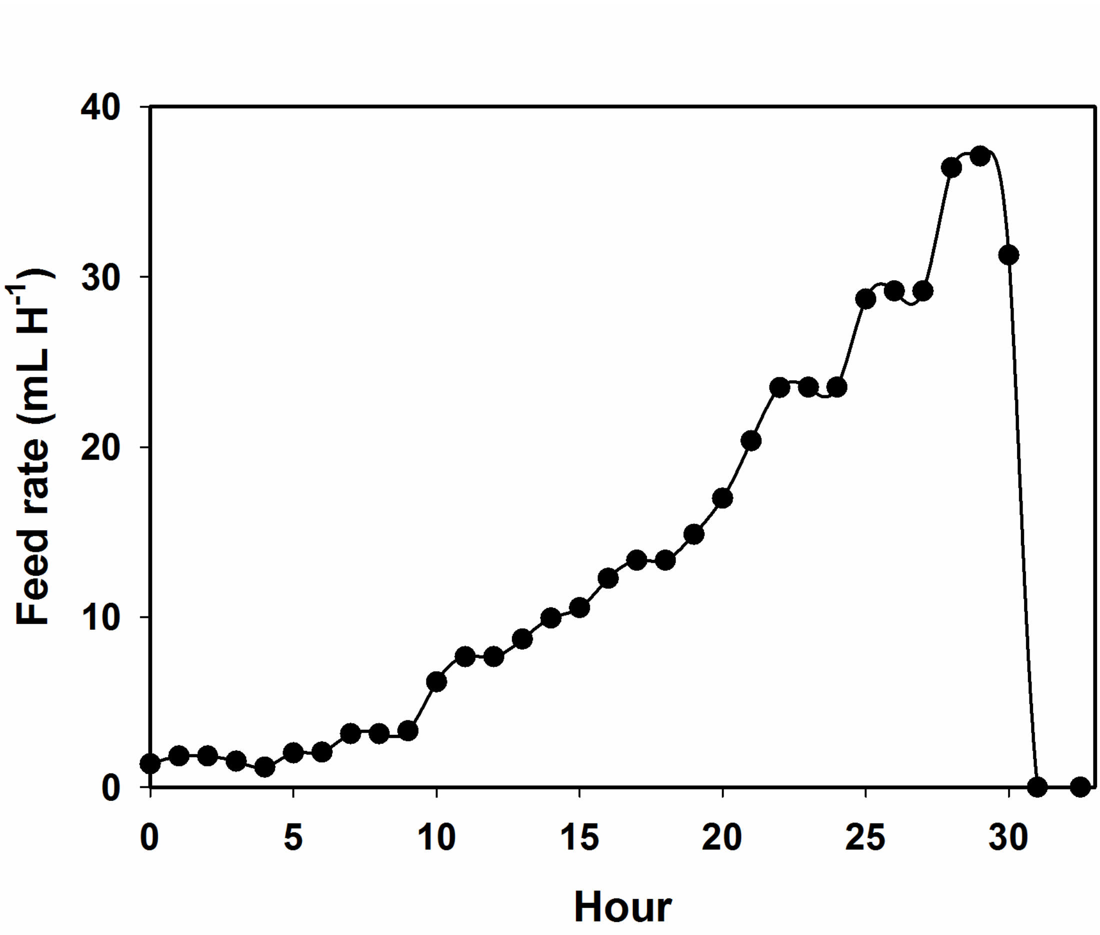

Figure S3. Feed rate control for *P. putida* KTΔ*pobA*Δ*pheA*Δ*trpE*Δ*hexR*/UA in fed-batch fermentation.

●, feed rate (ml h^{-1})

Table S2. Primers used in this study

Primers name	sequences	purpose
KTKO PobA P1	agcgcggtcaccaatgcttcgag	Knockout *pobA*
KTKO PobA P2	ccg**gaattc**tgtaaagcgcctcctgatcct	Knockout *pobA*
KTKO PobA P3	gccagcacctgcaccggcgcctgcaacctgagttttcat	Knockout *pobA*
KTKO PobA P4	ggcgccggtgcaggtgctggcttcgaggaagttgcctgaggg	Knockout *pobA*
KTKO PobA P5	cgc**ggatcc**aggcgctgttccaggttcgcta	Knockout *pobA*
KTKO PobA P6	aagcggtcgggacttaaggaggg	Knockout *pobA*
KTKO PheA P1	ttacgactacctggccatccc	Knockout *pheA*
KTKO PheA P2	c**ggaatt**ctgtcgcagttcggcctgatc	Knockout *pheA*
KTKO PheA P3	ccgccttcggctcctgttcggacat	Knockout *pheA*
KTKO PheA P4	gaacaggagccgaaggcggtgctttgat	Knockout *pheA*
KTKO PheA P5	gat**ggatcc**acgcctctaccccgctctgctcg	Knockout *pheA*
KTKO PheA P6	gcggaacaaggtggcattggacg	Knockout *pheA*
KTKO TrpE P1	ctggacggtaccctgatcgattc	Knockout *trpE*
KTKO TrpE P2	cg**gaattc**ctggaccagatgaagatcggta	Knockout *trpE*
KTKO TrpE P3	ggaagtctgctcttcttcgcggttcatgag	Knockout *trpE*
KTKO TrpE P4	atgaaccgcgaagaagagcagacttccgccagata	Knockout *trpE*
KTKO TrpE P5	tag**ggatcc**ggctacgacgagaagagc	Knockout *trpE*
KTKO TrpE P6	ggccagtgcctttgcccaatac	Knockout *trpE*
KTKO HexR P1	cgtcatcccagcgaagacctgt	Knockout *hexR*
KTKO HexR P2	cg***gaattc***atggcggctggacctgtg	Knockout *hexR*
KTKO HexR P3	acccatggaccgcgtgcgaaacatcgaggacgacgacctcaactg	Knockout *hexR*
KTKO HexR P4	tcagttgaggtcgtcgtcctcgatgtttcgcacgcggtccatgggt	Knockout *hexR*
KTKO HexR P5	cgc***ggatcc***ttcgcagatggccccatacacc	Knockout *hexR*
KTKO HexR P6	gcccaggtaatggtcaatgcggta	Knockout *hexR*
Ubic EcoRI Pf	gg**gaattc**taaggaggtaaccaaatgagccatccggccctg	Overexpress *ubiC*
Ubic HindIII Pr	gtcc**aagctt**tcagtacagcgggctgg	Overexpress *ubiC*
AroG SalI Pf	tga**gtcgac**tacccaaaagcca	Overexpress $aroG^{D146N}$
AroG HindIII Pr	ctc**aagctt**tcaaccgcga	Overexpress $aroG^{D146N}$

SEQ 234F	ctgtggtatggctgtgcaggt	Sequencing primer for SEVA serial plasmids
SEQ 234 R	ggacccctggattctcaccaa	Sequencing primer for SEVA serial plasmids
SEQ pEMG fwd2	ccccatgcgctccatcaagaaga	Sequencing primer for pEMG serial plasmids
SEQ pEMG rwd	cgactggaaagcgggcagtgag	Sequencing primer for pEMG serial plasmids

Note: restriction enzymes sites were highlighted in bold;

Codon optimized *ubiC* gene:

ATGAGCCATCCGGCCCTGACCCAGCTGCGTGCCCTGCGCTACTGCAAAGAGATCCCGGCCCTGGACCCGCAGC
TGCTGGATTGGCTGCTGCTGGAAGATAGCATGACCAAGCGCTTCGAGCAGCAGGGCAAGACCGTGAGCGTG
ACCATGATCCGCGAGGGCTTCGTGGAACAGAACGAGATCCCGGAAGAACTGCCGCTGCTGCCGAAAGAGAG
CCGCTACTGGCTGCGCGAGATCCTGCTGTGCGCCGACGGCGAACCGTGGCTGGCGGGTCGTACCGTGGTGCC
GGTGAGCACCCTGAGCGGTCCGGAACTGGCCCTGCAGAAGCTGGGCAAGACCCCGCTGGGCCGTTACCTGTT
CACCAGCAGCACCCTGACCCGTGACTTCATCGAGATCGGCCGTGATGCCGGCCTGTGGGGCCGTCGTAGCCG
TCTGCGCCTGAGCGGCAAGCCGCTGCTGCTGACCGAACTGTTCCTGCCGGCCAGCCCGCTGTACTGA

Codon optimized *aroG*^{D146N} gene:

ATGAACTACCAGAACGACGACCTGCGCATCAAGGAAATCAAGGAACTGCTGCCGCCGGTGGCCCTGCTGGAA
AAGTTCCCGGCCACCGAAAACGCCGCCAACACCGTGGCCCACGCCCGCAAGGCCATCCACAAGATCCTGAAG
GGCAACGACGACCGCCTGCTGGTGGTGATCGGCCCGTGCTCGATCCACGACCCGGTGGCCGCCAAGGAATAC
GCCACCCGCCTGCTGGCCCTGCGCGAAGAACTGAAGGACGAACTCGAGATCGTGATGCGCGTGTACTTCGAA
AAGCCGCGCACCACCGTGGGCTGGAAGGGCCTGATCAACGACCCGCACATGGACAACTCGTTCCAGATCAAC
GATGGTCTGCGCATCGCCCGCAAGCTGCTGCTGGACATCAACGACTCGGGCTTGCCGGCCGCCGGTGAATTTC
TGAACATGATCACCCCGCAGTACCTGGCCGACCTGATGTCGTGGGGGGCGATCGGTGCACGTACGACCGAAT
CGCAAGTCCACCGCGAACTGGCCTCGGGCCTGTCGTGCCCCGTGGGGTTTAAGAACGGCACCGACGGCACCA
TCAAGGTGGCCATCGACGCCATCAACGCCGCCGGCGCCCCGCACTGCTTCCTGTCGGTGACCAAGTGGGGCC
ACTCGGCCATCGTGAACACCTCGGGCAACGGCGACTGCCACATCATCCTGCGCGGCGGCAAGGAACCGAACT
ACTCGGCCAAGCACGTGGCCGAAGTGAAGGAAGGCCTGAACAAGGCCGGCCTGCCGGCCCAGGTGATGATC
GACTTCTCGCACGCCAACTCGTCGAAGCAGTTCAAGAAGCAGATGGACGTGTGCGCCGACGTGTGCCAACAG
ATCGCCGGCGGCGAAAAGGCCATCATCGGCGTGATGGTGGAATCGCACCTGGTGGAAGGCAACCAGTCGCT
GGAATCGGGCGAACCGCTGGCCTACGGCAAGTCGATCACCGACGCCTGCATCGGCTGGGAAGACACCGACG
CCCTGCTGCGCCAGCTGGCCAACGCCGTGAAGGCCCGCCGCGGCTGA
